# An Unrecognized Hazard in E-Cigarette Vapor: Preliminary Quantification of Methylglyoxal Formation from Propylene Glycol in E-Cigarettes

**DOI:** 10.3390/ijerph18020385

**Published:** 2021-01-06

**Authors:** Parham Azimi, Zahra Keshavarz, Marianne Lahaie Luna, Jose Guillermo Cedeno Laurent, Jose Vallarino, David C. Christiani, Joseph G. Allen

**Affiliations:** 1 Department of Environmental Health, Harvard T. H. Chan School of Public Health, Boston, MA 02115, USA; pazimi@hsph.harvard.edu (P.A.); zkeshavarz@hsph.harvard.edu (Z.K.); marianne.lahaieluna@mail.utoronto.ca (M.L.L.); memocedeno@mail.harvard.edu (J.G.C.L.); jvallari@hsph.harvard.edu (J.V.); dchris@hsph.harvard.edu (D.C.C.); 2 Occupational & Environmental Health Division, Dalla Lana School of Public Health, University of Toronto, Toronto, ON M5T 3M7, Canada

**Keywords:** e-cigarette, hazardous exposure, methylglyoxal, propylene glycol

## Abstract

Up to 95% of the liquid volume in an e-cigarette consists of propylene glycol. Previous research has shown that propylene glycol can generate diacetyl and formaldehyde when heated. New research shows that propylene glycol can also generate methylglyoxal, an alpha di-carbonyl compound recently shown to cause epithelial necrosis at even lower concentrations than diacetyl, the flavoring chemical associated with bronchiolitis obliterans (“Popcorn Lung”). We analyzed chemical emissions from 13 JUUL pod flavors. Diacetyl and methylglyoxal was detected in 100% of samples with median concentration (range) of 20 µg/m^3^ (less than limit of quantification: 54 µg/m^3^) and 4219 µg/m^3^ (677–15,342 µg/m^3^), respectively. We also detected acetaldehyde (median concentration: 341 µg/m^3^) and propionaldehyde (median concentration: 87 µg/m^3^) in all samples. The recent evidence that methylglyoxal is more cytotoxic to airway epithelial cells than diacetyl makes this an urgent public health concern. Current smokers considering e-cigarettes as a smoking cessation tool, and never users, who may be under the impression that e-cigarettes are harmless, need information on emissions and potential risks to make informed decisions.

## 1. Introduction

Reportedly, there are more than 13 million e-cigarette users in the US. Overall, 15.4% of adults aged more than 18 years had used an e-cigarette and 3.2% of them are regular e-cigarettes users based on National Health Interview Survey data [1]. The 2019 National Youth Tobacco Survey results show more than 5 million middle and high school students reporting having used e-cigarettes in the past 30 days and nearly one million reporting daily use [2]. There are also many users who are using e-cigarettes as a smoking cessation tool [3].

The U.S. e-cigarette market is expected to reach 16.5 billion USD by 2024 and the US e-cigarette brand JUUL is now reportedly holding nearly 75% of the market share in the US e-cigarette market [4]. E-cigarettes consist of a heating coil and e-cigarette liquid. When the user inhales, the heat coil is activated, generating a vapor that the user inhales. Propylene glycol is used as one of the predominant carrier fluids in many e-cigarettes, constituting 30–95% of the liquid in an e-cigarette by volume [5,6,7,8]. When heated, as in e-cigarettes, propylene glycol can generate secondary products. This potential for secondary product formation from heated propylene glycol was first raised around the issue of formaldehyde in e-cigarettes. Jenson et al. provided evidence of the previously unknown potential for e-cigarettes to generate formaldehyde [9] and several follow-up studies confirmed the generation of formaldehyde in e-cigarettes under typical conditions [8,10,11,12].

**Figure 1:**
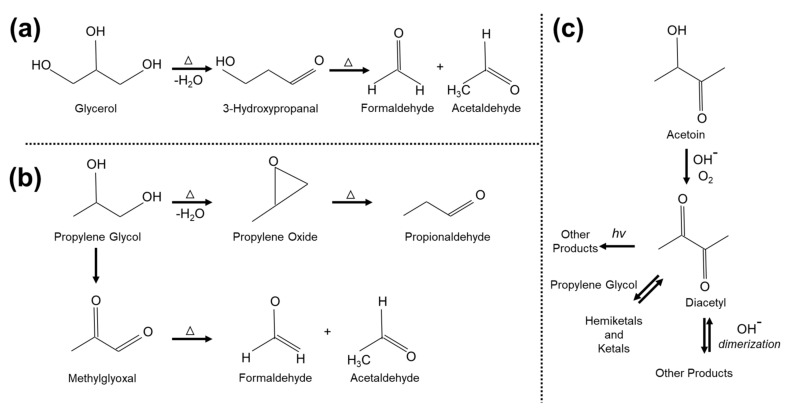
Proposed reaction mechanisms for formation of (**a**,**b**) formaldehyde, methylglyoxal, propionaldehyde, and acetaldehyde in e-cigarette puffs [11] and (**c**) diacetyl in e-cigarette liquids [13].

The potential for secondary product generation from propylene glycol extends beyond formaldehyde. In our previous research paper on formaldehyde in e-cigarettes [11], we included an illustration of the mechanism by which formaldehyde can be generated from heating propylene glycol (Figure 1a,b). In this figure, we also captured that propylene glycol can generate methylglyoxal and other toxic chemicals such as acetaldehyde and propionaldehyde. Moreover, in a later study, Vas et al. suggested that acetoin may be a precursor to diacetyl formation in e-cigarette liquids and proposed a reaction mechanism explaining the formation process as shown in Figure 1c [13]. In total, this body of research demonstrates that methylglyoxal, diacetyl, and other toxic carbonyl and di-carbonyls can be generated from e-cigarettes under typical heating coil temperatures.

Methylglyoxal is a major cell-permeant precursor of advanced glycation end-products (AGEs), which are associated with several pathologies including diabetes, aging and neurodegenerative diseases [14]. The theoretical potential of generating methylglyoxal is supported by actual testing of e-cigarettes, showing methylglyoxal being generated and inhaled by vapers [15,16,17,18]. The previously mentioned study by Talih et al. also reported detecting methylglyoxal in JUUL e-cigarettes. A separate study from Dator and Balbo which tested the saliva of vapers found that levels of methylglyoxal increased after vaping, further adding support to this hypothesis [19]. Diacetyl is a chemical that was found to be a prominent volatile constituent in butter flavoring and air in the microwave popcorn plant initially investigated by The National Institute for Occupational Safety and Health (NIOSH) because of health issues seen among the workers in microwave popcorn manufactures. Although the initial NIOSH studies were not able to definitely determine if diacetyl exposure contributed to lung disease as the workers were exposed to many materials beside diacetyl, several follow up studies have helped to clarify the role of diacetyl in substance toxicity [20,21,22]. Acetaldehyde toxicity has also been reviewed in several publications. Eye irritation has been reported in human volunteers exposed to acetaldehyde concentrations of as low as 50 ppm, while nose and throat irritation was also reported in individuals after exposure to acetaldehyde at concentrations typically greater than 100–200 ppm [23,24,25]. Limited information is available on the health effects of propionaldehyde. While no information is available on the acute (short-term), chronic (long-term), reproductive, developmental or carcinogenic effects of propionaldehyde in humans, studies on animals have shown propionaldehyde to have moderate acute toxicity from inhalation, oral and dermal exposures [26]. Specifically, it is shown that inhalation exposures to high levels of propionaldehyde caused anesthesia and liver damage in animals [27]. Herein, we investigated the emissions of methylglyoxal, diacetyl and other toxic carbonyl and dicarbonyls such as acetaldehyde and propionaldehyde from e-cigarettes and evaluated their potential health impacts.

## 2. Methodology

We selected 13 e-cigarette flavors representing the market-leading e-cigarette brand, JUUL, as listed in Table 1. Those 13 e-cigarette flavors were all available JUUL pod flavors that we could purchase from the JUUL and Amazon websites during the study. All the e-cigarette pods and devices were from the JUUL brand and they were purchased online in July 2019. The e-cigarette flavors were tested within two weeks of purchase. The selected flavors were nicotine-containing e-cigarettes with either 3% or 5% nicotine strength. The e-cigarettes consisted of disposable cartridges with a rechargeable battery. Similar to all JUUL products, the tested e-cigarettes used a ‘temperature-regulated’ system to avoid dry puff [28], which means users could not modify the temperature setting of the e-cigarettes to produce more power on the heating coil component. All e-cigarettes tested were automatic, used manufacturer-supplied batteries and were activated by the draw from an air pump puff.

All sampling activities were performed in Dr. David C. Christiani’s Laboratory in the Department of Environmental Health at Harvard T.H. Chan School of Public Health. Each e-cigarette was sampled for two seconds every minute during an approximate 4-h sampling period. Downstream smoke from the e-cigarettes was collected using a TE-2B Smoking Machine, Teague Enterprises (Woodland, CA, USA). The original air pump of the smoking machine was replaced with a more powerful air pump (MEDO/Nitto Kohki Co, VP0625 linear pump, Roselle, USA) that works continuously behind the electric air valve switch of the smoking machine to provide an appropriate combination of puff volume and duration for activating the e-cigarettes and produce rectangular wave puff profiles. The setup was configured to sample from two e-cigarettes, altering a continuous airflow between samples and room air (i.e., from e-cigarette sample 1 for 2 s, to room air for 28 s, to e-cigarette sample 2 for 2 s, and again to room air for 28 s) to reduce the experiment duration. 

The Puff flow rates were set using SKC low flow inline control valves to approximately the minimum flow required to activate the e-cigarettes. The airflow was measured using a TSI 4146 Flow Calibrator, Shoreview, USA, at the start and the end of the sampling periods for each sampling branch. The TSI 4146 Flow Calibrator was placed temporarily between impingers and the inline control valves for a limited time (i.e., a few seconds) to minimize any potential damage related to passing contaminated air through the calibrator, and the monitor readings were checked and calibrated several times during the study. The airflow was also continuously monitored during the experiment periods using an Omron D6F-P0001A1 flow sensor (Kyoto, Japan), while the readings were recorded by a HOBO UXT120 006 data logger (Onset Corp, Bourne, MA, USA). The Omron D6F-P0001A1 flow sensors were used solely to confirm the puff duration and consistency of the airflow during the sampling session. The smoke stream was passed through a 25 mL Zefon glass midget impinger filled with 20 mL of ultra-pure water as the collection liquid immediately after being emitted from the e-cigarettes.

**Figure 2:**
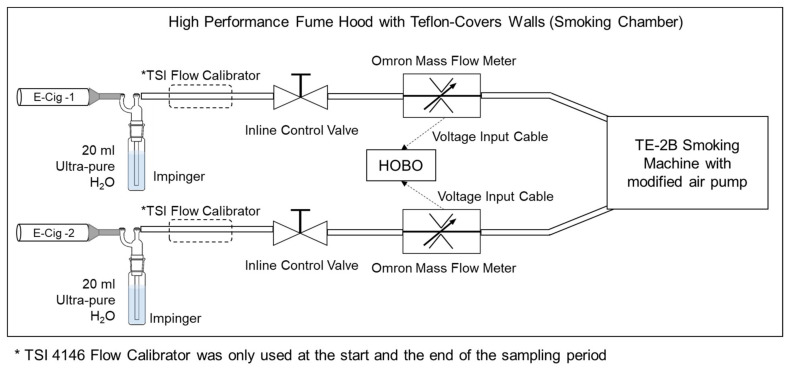
Schematic diagram of the experiment setup.

The average airflows ranged between 0.44 and 0.98 L per minutes (LPM) and consequently, the puff volumes were estimated between 14 cm^3^ and 33 cm^3^, and the sampling duration was approximately 4 h ranging between 240 and 251 min. Similar experiment setups were used previously in other peer-reviewed journal publications for measuring the emissions of toxic chemicals from e-cigarettes [11,29]. Figure 2 demonstrates a schematic diagram of the setup and setup characteristics of the tested e-cigarette flavors are summarized in Table 1. 

It is noticeable that the average sampling flow was varied for each test depending on the final volume of collection liquid in impingers, deployed e-cigarettes (i.e., a total of four identical JUUL e-cigarettes were used during the study), and other factors, which were not immediately obvious to us. At the end of each test, the remaining collection liquid in impingers (usually less than 20 mL as some of the liquid evaporated during the sampling period) was transferred to a liquid media sampler plastic tube, the liquid volume was increased back to 20 mL by adding ultra-pure water, and the samples were immediately stored in a laboratory freezer at −20 °C.

The liquid media sampler plastic tubes containing the collection liquid samples were wrapped in aluminum foil sheets, packed with ice packs in an insulated bag, and sent to Labstat International Inc. (“*Labstat*”), an independent center that specializes in analytics related to tobacco and tobacco products in Canada (Kitchener, Ontario), via an overnight shipping service. The laboratory confirmed that the shipment were received without any physical damage to the boxes and individual test items were normal in appearance. The laboratory determined the concentrations of selected carbonyls in the e-cigarette condensate samples using O-2,3,4,5,6-(Pentafluorobenzyl) hydroxylamine hydrochloride (PFBHA) derivatization and gas chromatography/mass spectrometry analysis [30,31,32,33]. The selected carbonyls and di-carbonyls included acetaldehyde, acetoin, isobutyraldehyde, propionaldehyde, 2,3-butanedione (diacetyl), 2,3-heptanedione, 2,3-hexadione, 2,3-pentanedione and methylglyoxal.

For each tested flavor, 5.0 mL of the provided liquid was aliquoted and processed for analysis of mainstream vapor samples. 2.5 g of liquid test sample was mixed with 5 mL of Type I water in a 10 mL glass tube. After adding 100 µL of mixed internal standard solution containing D_4_-acetaldehyde, D_6_-acetone, D_5_-MEK, and D_6_-2,3-butanedione and 1000 µL of PFBHA (20 mg/mL) aqueous solution, the glass tube was capped and placed in the dark for derivatization for 24 ± 4 h. After derivatization with PFBHA, 5 drops of 18N H_2_SO_4_ were added and the PFBHA derivatives were extracted with 2 mL of toluene. The toluene extract was transferred to an autosampler vial for gas chromatography/mass spectrometry (GC/MS) analysis. The PFBHA derivatives of the target carbonyls were separated on a 30 m × 0.25 mm × 0.25 µm film thickness RTX-5 ms column and then quantified by a mass spectrometer using selected ion monitoring (SIM) mode. Analytical values reported on a μg/mL basis and converted to concentrations in μg/m^3^ of air using the recorded airflow rates and sample durations.

For quality control, in addition to 13 tested flavors, we collected one blank (ultra-pure water) and one duplicate sample (i.e., Virginia Tobacco flavor with 5% nicotine). The blank sample underwent the same storage and shipping process as the other e-cigarette flavors to identify potential contaminants interfering with the sampling results during the process. To test the recovery rate of the adopted measurement methods, the laboratory added a known amount of surrogate compounds (i.e., chemicals similar to the target analytes in chemical composition and behavior, but which are not expected to be present in the sample) to three collection liquid samples during the preparation stage of the analysis. The percent recovery for each surrogate compound was measured and reported by the laboratory. Table 2 summarizes the laboratory’s limit of detection (LOD), limit of quantification (LOQ), and recovery rates for selected carbonyls and di-carbonyls in the collection solution.

Briefly, for LOD and LOQ determinations, concentrations of a wide range of flavor compounds (usually >50) potentially used in e-liquids were measured several times (usually >20 times) in a semi-quantification process and the standard deviation of these results was calculated. The LOD was calculated as ~3 times the standard deviation of the response divided by the slope of the nicotine calibration curve and LOQ was calculated as ~10 times the standard deviation of the response divided by the slope of the nicotine calibration curve. The reported recovery rates are the recovery rates of surrogate chemicals added to all environmental samples, blanks and QC samples in the analytical batch during the preparation stage of the analysis in Labstat. Surrogates are chemicals that are similar to the target analyte(s) in chemical composition and behavior, but which are not expected to be present in the sample. Surrogates are used to monitor analytical performance, especially extraction efficiency, purging efficiency (for volatiles) and possible matrix effects [34].

Table 2 demonstrates the average recovery percentages of surrogate compounds of the selected carbonyl and di-carbonyls reported by the laboratory. It is noticeable that several recovery rates were relatively higher than regular acceptable values (i.e., ±30%) [34]. However, as the laboratory could not find an assignable cause for the high recovery rates, the test result was assumed to be a legitimate member of the data set and was included in all subsequent calculations. Moreover, in this study, we used the originally reported results provided by the laboratory without applying correction factors based on the recovery rates, as the laboratory only measured the recovery rates for three out of 15 samples.

Finally, we estimated the average concentration of detected compounds in e-cigarette puffs (${\bar{C}}_{puff,i}$) from Equation (1):

(1) $${\bar{C}}_{puff,i}=\frac{C_{impinger,i}\times V_{sample}}{{\bar{F}}_{sampling}\times T_{sampling}\times R_{sampling}}$$

where: ${\bar{C}}_{puff,i}$: Average concentration of compound *i* in e-cigarette puffs (µg/m^3^); $C_{impinger,i}$: Laboratory-measured concentration of the compound *i* in impingers’ liquids after sampling (µg/cm^3^); $V_{sample}$: Volume of impinger sample liquid (for all samples was 20 cm^3^); ${\bar{F}}_{sampling}$: Average sampling flow (m^3^/min); $T_{sampling}$: Total measurement time (min); $R_{sampling}$: Ratio of sampling time to total measurement time (for all samples was 1/30).

## 3. Results

**Figure 3:**
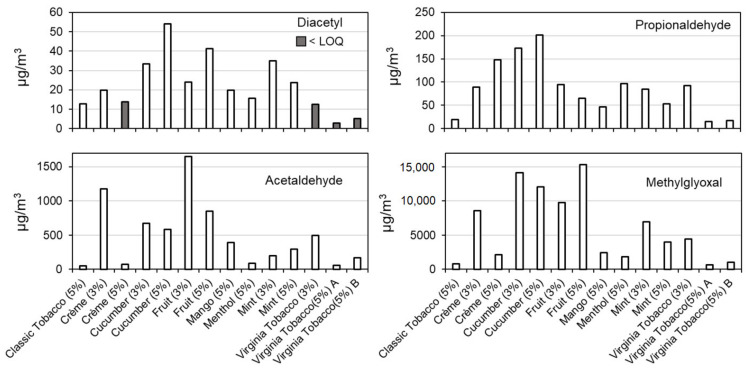
Concentration of selected carbonyls and di-carbonyls in e-cigarette puffs. LOQ: limit of quantification.

Concentrations of acetaldehyde, propionaldehyde, methylglyoxal and diacetyl in e-cigarettes puffs are presented in Figure 3. Methylglyoxal, diacetly, acetaldehyde, and propionaldehyde were all detected above the LOD in 100% of samples. For four collection liquid samples in which the concentration of diacetyl were above LOD but below LOQ, we reported the laboratory assigned concentrations, which were 0.003 µg/mL, 0.003 µg/mL, 0.001 µg/mL, and 0.002 µg/mL for 5-percent-nicotine Crème, 3-percent-nicotine Virginia Tobacco, 5-percent-nicotine Virginia Tobacco (sample A) and 5-percent-nicotine Virginia Tobacco (sample B) e-cigarettes, respectively.

The concentration of methylglyoxal in the tested flavored e-cigarette puffs was highest with approximately an order of magnitude higher than the second emitted compound, acetaldehyde. The concentration of emitted methylglyoxal was highest for Fruit and Cucumber flavors with values between ~10,000 µg/m^3^ and ~15,000 µg/m^3^. Tobacco flavoring e-cigarettes with 5% nicotine strength including Classic and Virginia Tobacco had the lowest emissions of methylglyoxal among tested flavors.

The emissions of acetaldehyde were lower than for methylglyoxal. Similar to methylglyoxal emissions, Fruit (3% and 5% nicotine strength), Cucumber (3% and 5% nicotine strength) and 3-percent-nicotine Crème e-cigarette flavors were the top five acetaldehyde emitters among other tested flavors. This is in line with the proposed reaction mechanism for the formation of acetaldehyde demonstrated in Figure 1, which suggests that acetaldehyde could form as a byproduct of methylglyoxal. The acetaldehyde concentrations in the e-cigarette puffs varied between ~50 and ~1650 µg/m^3^ for Classic Tobacco (5% nicotine strength) and Fruit (3% nicotine strength) e-cigarette flavors, respectively.

The concentrations of propionaldehyde and diacetyl were significantly lower than the other two major compounds. The top three propionaldehyde emitters among the tested flavors were 5- and 3- percent-nicotine Cucumber, and 5-percent-nicotine Crème e-cigarettes with estimated concentrations of 201, 173, and 148 µg/m^3^ in the e-cigarette puffs, respectively. Similar to methylglyoxal and acetaldehyde the propionaldehyde emissions were lowest for 5-percent-nicotine tobacco flavoring e-cigarettes with concentrations less than 20 µg/m^3^ in the e-cigarette puffs. The concentration of diacetyl in tested e-cigarette puffs varied between 3 and 54 µg/m^3^ for 5-percent-nicotine Virginia Tobacco and Cucumber flavors, respectively. As demonstrated in Figure 3, the estimated concentration of diacetyl in four e-cigarette puffs were considered to be less than LOQ and above LOD. This is because the concentration of diacetyl in their collection liquid samples were below the laboratory LOQ, as shown in Table 3.

The laboratory analysis measured the concentrations of contaminants in the collection liquid below LOD for acetoin, 2,3-Pentanedione, 2,3-Hexanedione and 2,3-Heptanedione and between LOD and LOQ for Isobutyraldehyde. The concentrations of selected aldehydes in the blank sample remained below LOD for most of the chemicals except acetaldehyde, propionaldehyde and isobutyraldehyde, which were between LOD and LOQ.

## 4. Discussion

### 4.1. Public Health Concerns

The health concerns regarding electronic nicotine delivery systems and particularly e-cigarettes started almost immediately after their introduction to the market in the United States in 2007 [35,36,37,38,39]. Diacetyl was among the first chemicals investigated for its potential adverse health impacts because of previous studies showing association of diacetyl exposures with bronchiolitis obliterans (“Popcorn Lung” disease) [22,40]. Inhalation and toxicological studies in rodents suggest that chronic exposure to diacetyl can cause fibrous scarring of lungs [21,41] rapid growth of lymphocytes in lungs [42] and different types of pulmonary fibrosis, which is a type of irreversible and progressive lung disease [43]. Herein, we demonstrated the presence of diacetyl in e-cigarette emissions above LOD and LOQ concentrations depending on the flavoring chemicals used in e-cigarette liquids. Several existing studies also detected diacetyl in e-cigarette emissions [11,29,44,45] and liquids [46,47].

NIOSH suggested 8-h recommended exposure limit (REL) of 5 ppb and 15-min ceiling short-term exposure limits (STEL) of 25 ppb for adult workers for diacetyl and its structurally similar replacement, 2,3-pentanedione. However, there are no health-based standards for diacetyl inhalation for the general public, and no standards for children [48]. Egilman et al. in 2011 proposed various safe 8-h exposure levels for diacetyl based on four disease outcomes [49]. The safe levels were varied from < 1 to 8 ppb based on a revised analysis of toxicology excellence for risk assessment and other sources. Assuming diacetyl as an ideal gas with a molecular weight of 86 g/mol, the measured concentration of diacetyl in e-cigarette fumes in this research work were from 0.9 ppb (lower than LOQ) for Virginia Tobacco e-cigarette with 5% nicotine to 15.4 ppb for Cucumber e-cigarette with 5% nicotine. This means 8 and 10 out of 13 tested e-cigarette flavors emit diacetyl higher than the NIOSH 8-h REL and the safe exposure levels of diacetyl proposed by Egilman et al., respectively. For example, less than one-hour exposure to Mint, Cucumber, and Fruit flavoring e-cigarette emissions per day would exceed the average daily diacetyl exposures by more than the lower ranges of safe exposure levels proposed by Egilman et al.

Several studies also reported emissions of methylglyoxal from e-cigarettes [16,18,50]. The confirmation of methylglyoxal in e-cigarette emissions takes on new importance for e-cigarette users when considering the potential toxicity. A study published in 2019 by Hubbs et al., which included some of the investigators who evaluated the initial Popcorn Lung cases in the early 2000s, reviewed the toxicity of diacetyl and other related alpha-di-carbonyls, including methylglyoxal [51]. Beyond reviewing the mechanisms of alpha-di-carbonyl toxicity, the authors undertook toxicological testing of methylglyoxal, a 3-carbon alpha-di-carbonyl (Diacetyl and 2,3-pentandeione are 4- and 5-carbon alpha carbonyls, respectively). The authors examined necrosis of respiratory and transitional epithelium of the mouse and report, ‘methylglyoxal, a 3-carbon a-carbonyl compound, is not only cytotoxic, it is actually more cytotoxic than diacetyl, a 4-carbon a-di-carbonyl compound.’ Specifically, they found that methylglyoxal ‘caused dose-dependent necrosis of the respiratory and transitional epithelium’.

Several other studies also demonstrated the adverse health outcomes of exposure to methylglyoxal. Nigro et al. showed that increased concentration of methylglyoxal impairs the action of insulin on vascular endothelium both in vitro and in vivo, leading to an imbalance between nitric oxide and endothelin-1 production [52]. A literature review study from same group in 2017 showed that methylglyoxal accumulation has harmful effects on vascular function, by inducing insulin-resistance, hypertension, atherosclerosis, neurodegenerative disease and diabetic microvascular complications [53]. Another literature review study summarized the potential adverse health outcomes of exposures to methylglyoxal [54] including a potential association with diabetic polyneuropathy [55], production of imidazopurinones, nucleotide adducts—which were considered as major types of endogenous DNA damage [56]—and generation of neuropathic pain [57].

Acetaldehyde and propionaldehyde were two other toxic carbonyls detected in all tested e-cigarette flavors with concentrations above the laboratory limits of quantifications. Acetaldehyde [10,11,16,18,44,50,58] and Propionaldehyde [10,11,44,50] were detected in several existing studies determining the chemical compounds generated by e-cigarettes. Researchers demonstrated that acetaldehyde induces DNA-protein crosslinks, which are known to cause lethal consequences for a cell if unrepaired. Therefore, exposure to acetaldehyde is expected to interfere with DNA metabolic process such as replication, repair, recombination, transcription and chromatin remodeling [59], and induce mucin 2 (MUC2) as representing the major secretory mucin of the small and large intestines [54]. No studies in humans are available for propionaldehyde; however, based on the information provided from animal studies, the most likely adverse human health effects that would be anticipated from exposure to propionaldehyde would be primarily respiratory tract irritation and secondarily cardiovascular perturbations [60].

### 4.2. Limitations

The emission measurements of methylglyoxal, diacetyl, and other toxic carbonyl and dicarbonyls such as acetaldehyde and propionaldehyde from e-cigarettes presented in this study should be considered as preliminarily quantification of secondary formation of toxic compounds from propylene glycol in e-cigarettes. The measurements also demonstrate a comparison across the tested e-cigarette pod liquids. The reasons for considering the presented results preliminarily are: (i) we did not use a sample collection efficiency metric, such as reference cigarettes, in our measurement approach and (ii) the average collected puff volume in this study (i.e., 22.3 mL) was lower than regular human puff volume (i.e., 55 mL) to provide enough time for chemical components to resolve in impingers’ liquids. Future studies should be performed to quantify the concentrations of the secondary-formed chemicals under standard conditions such as ISO 20768 [61].

There is limited information about human health outcomes of exposures to methylglyoxal, acetaldehyde and propionaldehyde at measured concentration levels in this study, although many in vitro and animal studies suggest potential health risks associated with these chemicals. More studies in this field would provide necessary information for generating new guidelines and standards and updating existing ones for safe levels of exposures to methylglyoxal, acetaldehyde and propionaldehyde for human individuals.

It is noticeable that there is a large variation in the recovery rates reported by the laboratory (i.e., Labstat). While, the regular acceptable values of recovery rate is 100% ± 30% [34], we observed in some samples the average reported recovery rate was more than 150%. This could mean that the concentration of chemical compounds in cases with high recovery rate is most probably overestimated. However, it is important to note that even in cases where high recovery rates were reported, the measured concentrations were still above the laboratory limit of detection.

Finally, the measured emission rates of methylglyoxal, diacetyl, acetaldehyde and propionaldehyde were significantly different across the tested e-cigarette flavors. In this study, we did not measure the PG/VG ratio of the tested pods; however, other existing studies reported the PG ratio of JUUL flavored pods is 30% by volume [8,62]. If we assume that PG/VG is constant across all JUUL flavored pods, it means that other factors should have influenced the variation in emissions of secondary toxic compounds formed from propylene glycol in e-cigarettes. It is not fully clear what these factors are, and further investigation is needed.

## 5. Conclusions

Current ingredient labels on e-cigarette packages do not reveal the full potential of respiratory toxicants that users may be inhaling. This includes chemicals that are added intentionally, like flavoring chemicals and other compounds [29,63] as well as secondary compounds formed from e-cigarette ingredients during smoking. In addition to potential exposure to alpha-di-carbonyls like diacetyl and 2,3-pentanedione, studies are demonstrating that exposure to methylglyoxal through secondary formation from propylene glycol is likely widespread. The recent evidence that methylglyoxal is more cytotoxic than diacetyl, an alpha-carbonyl linked to bronchiolitis obliterans, makes this an urgent public health concern.

## Figures and Tables

**Table 1:** Sampling characteristics of tested flavors.

Tested JUUL Pods	Ave. Sampling Flow (Start-End) (LPM)	Sampling Time (min)	Air Sampled per Puff (cm^3^)	Total Air Sampled (L)
Classic tobacco (5%)	0.88 (0.80–0.96)	240	29.3	7.036
Crème (3%)	0.41 (0.32–0.51)	245	13.8	3.377
Crème (5%)	0.52 (0.41–0.64)	240	17.5	4.188
Cucumber (3%)	0.53 (0.52–0.54)	244	17.7	4.315
Cucumber (5%)	0.62 (0.50–0.74)	240	20.6	4.952
Fruit (3%)	0.44 (0.43–0.45)	251	14.8	3.715
Fruit (5%)	0.92 (0.92–0.93)	245	30.8	7.546
Mango (5%)	0.98 (0.96–1.00)	243	32.6	7.914
Menthol (5%)	0.78 (0.77–0.80)	245	26.1	6.403
Mint (3%)	0.61 (0.58–0.63)	245	20.2	4.945
Mint (5%)	0.52 (0.51–0.53)	245	17.3	4.230
Virginia Tobacco (3%)	0.53 (0.50–0.55)	240	17.6	4.212
Virginia Tobacco (5%) A	0.77 (0.73–0.80)	240	25.6	6.140
Virginia Tobacco (5%) B	0.85 (0.84–0.86)	240	28.3	6.800

**Table 2:** Laboratory’s limit of detection (LOD), limit of quantification (LOQ) and recovery rates for all selected carbonyls and di-carbonyls.

Carbonyls/Di-Carbonyls	LOD (µg/mL) *	LOQ (µg/mL) *	Ave. Recovery Rates (Range) **
Acetaldehyde	0.005	0.017	121 (113–130)
Propionaldehyde	0.002	0.008	150 (102–176) ^†^
Isobutyraldehyde	0.001	0.003	142 (102–168) ^†^
Acetoin	0.003	0.011	167 (120–201) ^†^
2,3-Butanedione (Diacetyl)	0.001	0.003	105 (101–108)
2,3-Pentanedione	0.002	0.006	92 (89–96)
2,3-Hexanedione	0.002	0.006	141 (84–220) ^†^
2,3-Heptanedione	0.002	0.008	88 (69–120)
Methylglyoxal	0.001	0.003	149 (107–175) ^†^

* Contaminant concentration in the collection liquid; ** The recovery rates were not applied to the laboratory results; ^†^ The laboratory could not assign a cause for high recovery rate.

**Table 3:** Carbonyl and Di-carbonyl contents of e-cigarette collection liquids.

Sample	Acetaldehyde	Propionaldehyde	Methylglyoxal	Diacetyl
E-cigarette Flavor (Nicotine Strength)	[µg/mL]	[µg/mL]	[µg/mL]	[µg/mL]
Blank	<0.017 but ≥0.005	<0.008 but ≥0.002	<0.001	<0.001
Classic tobacco (5%)	0.031	0.011	0.284	0.005
Crème (3%)	0.213	0.019	1.45	0.003
Crème (5%)	0.030	0.035	0.443	<0.003 but ≥0.001
Cucumber (3%)	0.159	0.041	3.05	0.007
Cucumber (5%)	0.158	0.054	3.00	0.013
Fruit (3%)	0.320	0.022	1.82	0.004
Fruit (5%)	0.334	0.028	5.79	0.016
Mango (5%)	0.167	0.022	0.978	0.008
Menthol (5%)	0.041	0.035	0.597	0.005
Mint (3%)	0.063	0.025	1.73	0.009
Mint (5%)	0.075	0.015	0.841	0.005
Virginia Tobacco (3%)	0.118	0.023	0.940	<0.003 but ≥0.001
Virginia Tobacco (5%) A	0.034	0.009	0.230	<0.003 but ≥0.001
Virginia Tobacco (5%) B	0.065	0.009	0.316	<0.003 but ≥0.001

## Data Availability

Data is contained within the article.
